# Pretreatment and enzymatic process modification strategies to improve efficiency of sugar production from sugarcane bagasse

**DOI:** 10.1007/s13205-016-0446-2

**Published:** 2016-06-07

**Authors:** Siddhartha Pal, Shereena Joy, Kalpana D. Trimukhe, Pramod S. Kumbhar, Anjani J. Varma, Sasisanker Padmanabhan

**Affiliations:** 1 Praj Matrix R&D Center, Urawade, Pune, Maharashtra India; 2 Department of Technology, Savitribai Phule Pune University, Ganeshkhind, Pune, Maharashtra India; 3 Polymer Science and Engineering Division, CSIR-National Chemical Laboratory, Pune, Maharashtra India; 4 Central University of Haryana, Post-Pali District, Mahendergarh, Haryana 123029 India

**Keywords:** Pilot-scale pretreatment, Dilute acid treatment, High-solid enzymatic hydrolysis, Pressure filtration, Solid–liquid separation

## Abstract

Pretreatment and enzymatic hydrolysis play a critical role in the economic production of sugars and fuels from lignocellulosic biomass. In this study, we evaluated diverse pilot-scale pretreatments and different post-pretreatment strategies for the production of fermentable sugars from sugarcane bagasse. For the pretreatment of bagasse at pilot-scale level, steam explosion without catalyst and combination of sulfuric and oxalic acids at low and high loadings were used. Subsequently, to enhance the efficiency of enzymatic hydrolysis of the pretreated bagasse, three different post-pretreatment process schemes were investigated. In the first scheme (Scheme 1), enzymatic hydrolysis was conducted on the whole pretreated slurry, without treatments such as washing or solid–liquid separation. In the second scheme (Scheme 2), the pretreated slurry was first pressure filtered to yield a solid and liquid phase. Following filtration, the separated liquid phase was remixed with the solid wet cake to generate slurry, which was then subsequently used for enzymatic hydrolysis. In the third scheme (Scheme 3), the pretreated slurry was washed with more water and filtered to obtain a solid and liquid phase, in which only the former was subjected to enzymatic hydrolysis. A 10 % higher enzymatic conversion was obtained in Scheme 2 than Scheme 1, while Scheme 3 resulted in only a 5–7 % increase due to additional washing unit operation and solid–liquid separation. Dynamic light scattering experiments conducted on post-pretreated bagasse indicate decrease of particle size due to solid–liquid separation involving pressure filtration provided increased the yield of C6 sugars. It is anticipated that different process modification methods used in this study before the enzymatic hydrolysis step can make the overall cellulosic ethanol process effective and possibly cost effective.

**Electronic supplementary material:**

The online version of this article (doi:10.1007/s13205-016-0446-2) contains supplementary material, which is available to authorized users.

## Introduction

In recent times, various pilot and demonstration scale operations have been carried out for the biochemical conversion of lignocellulosic (LC) biomass to fuel ethanol and other essential commodity biochemicals (Klein-Marcuschamer and Blanch 2015; Agrawal et al. 2015; Larsen et al. 2012). Typically, most of the large-scale LC ethanol production processes (pilot or demonstration scale plants) involve high-solid pretreatment followed by enzymatic hydrolysis to yield desirable sugars that can subsequently be fermented to ethanol or other biochemicals (Modenbach and Nokes 2013; Kristensen et al. 2009). It has been widely acknowledged that both pretreatment and enzymatic hydrolysis steps contribute significantly (~70 %) to the overall process economics and energy (Klein-Marcuschamer and Blanch 2015; Larsen et al. 2012; Macrelli et al. 2012). This is because effective pretreatment strategies overcome the recalcitrant nature of biomass and provide amenable substrate for the enzymatic hydrolysis. Similarly, effective strategies for enzymatic hydrolysis of pretreated materials provide better yield of sugar, ultimately contributing to high ethanol concentration following fermentation (Chundawat et al. 2011; Wyman et al. 2011).

Use of dilute acid (DA), hot-water and steam explosion (SE) for the pretreatment of LC biomass is considered relatively cost effective (Geng et al. 2015). These treatments highly solubilize xylose in monomeric or oligomeric form or a mixture of both in the aqueous phase (Geng et al. 2015; Guo et al. 2012; Hernandez et al. 2012). Furthermore, these treatments also keep the cellulose and lignin parts intact in the solid phase, where the former is subsequently converted to monomeric glucose sugars using cellulase enzymes.

In DA or SE process, high-solid (>15 %) pilot-scale pretreatment is carried out followed by enzymatic hydrolysis (Larsen et al. 2012; Modenbach and Nokes 2013; Kristensen et al. 2009; Geng et al. 2015). After pretreatment, the slurry is usually filtered to yield a solid phase (lignin and cellulose) and liquid phase (xylose or xylo-oligomers, HMF, furfural and phenolics), which is commonly denoted as pre-hydrolyzate or aqueous stream (Zhang et al. 2015). In most of the published studies, the separated liquid phase is then subsequently fermented directly to ethanol without any preconditioning (Macrelli et al. 2012). With regard to the solid part that remains after filtration, various processes, such as separate enzymatic hydrolysis and subsequent fermentation (SHF) and simultaneous saccharification and fermentation (SSF), are possible for the hydrolysis of cellulose to sugars and subsequent fermentation to ethanol (Wingren et al. 2003). In some studies, solid fraction is further washed extensively with water to remove the inhibitors, and then hydrolyzed and fermented separately or together with the prehydrolyzate (Ioelovich and Morag 2012; Xue 2011). All these processes require additional process water in the overall scheme of bioethanol production, leading to lower ethanol concentration and more energy input for distillation (Larsen et al. 2012; Macrelli et al. 2012). Besides, generally, in laboratory-scale processes, solid loading of 5–10 % is used in enzymatic hydrolysis to achieve acceptable yields of sugar and ethanol (Wingren et al. 2003; Ioelovich and Morag 2012). Therefore, new process methods that enhance the yield of fermentable sugars by enzymatic hydrolysis are highly desirable as higher sugar concentration results in higher ethanol concentration (4–5 w/w %) leading to lower energy requirements for ethanol distillation (Macrelli et al. 2012; Zacchi and Axelsson 1989).

To the best of our knowledge, only few studies have been reported on the various process scenarios for cellulose and hemicellulose hydrolysis using washed and unwashed sugarcane bagasse (SB) at a higher substrate loading and an enzyme concentration usually required for a large-scale process. Hence, the primary goal of this study is to explore various posttreatment methods to enhance enzymatic hydrolysis efficiency of bagasse. The first process method exploits whole-slurry hydrolysis concept (Scheme 1), where the pretreated slurry is used directly for enzyme hydrolysis, without any modifications such as washing or solid–liquid separation. The second process, denoted as reslurried hydrolysis (Scheme 2), uses pressure filtration technique to separate the solid and liquid phases from the pretreated slurry. The separated aqueous phase is remixed with the solid phase (wet filter cake) to form slurry, which is then used for enzyme hydrolysis. The third method uses wet cake hydrolysis scheme (Scheme 3), where both washing and pressure filtration were conducted on the pretreated slurry. The solid part of the filtration is further used for the enzymatic hydrolysis.

The streams required for this comparative study were obtained from pilot-scale treatments conducted at different values of combined severity factor (CSF), which is a function of acid dose, temperature, and retention time (Schell et al. 2003; Zhang et al. 2011, 2013). The three types of pretreatment methods used in this study are (1) dilute mixed-acid treatment at high severity (CSF 1.55), (2) mild mixed-acid treatment at intermediate severity (CSF 1.43) and (3) SE without catalyst at low severity (CSF 0.7). Sulfuric acid is chosen due to its high acidity, low cost, and well-known efficiency in most of the pretreatments (Todd and Wyman 2005). Oxalic acid is a strong but relatively non-corrosive dicarboxylic acid that is useful for xylan hydrolysis (Zhang et al. 2013).

## Experimental methods and analytical procedures

### Materials

SB was provided by Om Sai Chemicals, Maharashtra, India, with approximately 30–35 % moisture. It was stored under ambient conditions and used as such without any further drying. Before feeding into the pilot-scale reactor, SB was shredded in a hammer mill in the size range of 15–25 mm. Following is the composition of SB on dry basis: cellulose (40.3 ± 2 %), xylan (21.3 ± 0.7 %), arabinan (2.2 ± 0.1 %), acid-insoluble lignin (19.3 ± 0.8 %), acid-soluble lignin (3.9 ± 0.2 %), ash (2.6 ± 0.15 %), proteins (3.0 ± 0.1 %), acetate (2.9 ± 0.1 %) and extractives (5.0 ± 0.4 %).

### Compositional analysis methods

The composition of the raw material, pretreated solid and the oligomer analysis of the filtrate post-pretreatment were determined using the National Renewable Energy Laboratory (NREL) analytical procedure (Sluiter et al. 2008a, b, 2012).

The composition of pretreated slurry, post-enzymatic hydrolysis slurry, and post-co-fermentation slurry (fermented wash) was determined by measuring glucose, xylose, arabinose, acetic acid, hydroxymethylfurfural (HMF) and furfural in Aminex^®^ column (HPX-87H 300 × 7.8 mm BioRad column) with a flow rate of 0.6 mL/min, temperature of 55 °C, and mobile phase of 0.005 M sulfuric acid. The post-co-fermentation slurry was also measured for ethanol concentration using the same column.

The filtered samples of hydrolyzate (from pretreatment) were restored to a final sulfuric acid concentration of 4 w/w %, autoclaved at 121 °C for 1 h, and centrifuged for high-performance liquid chromatography (HPLC) determination of monomer sugars. The total oligomeric content was determined as the difference between the amounts of monomer obtained after post-acid hydrolysis, following the correction of losses, and before acid hydrolysis.

The total phenolic content of the sample was measured with spectrophotometrical method according to Folin–Ciocalteu reagent redox reaction (Singleton et al. 1999). The absorbance was measured after 30 min at room temperature at 700 and 725 nm. Calibration curves based on vanillin were used for quantification because gas chromatography–mass spectrum (GC–MS) indicated vanillin being most present in the acid-treated slurry.

### Pretreatment methods

#### Continuous pretreatment

Pretreatments were done using one ton per day (TPD) continuous pilot-scale horizontal screw-type reactor. The reactor set up is similar to the one described in the work of Schell et al. (2003). The total solid handling capacity of this reactor is up to 22 % (w/w). Size reduced SB was fed to the pretreatment reactor using a belt conveyor. This reactor was part of a reactor feeder blow tank system operating at a feed rate of 30 kg on dry weight basis/hour. The system is supplied with constant acid, steam pressure, and temperature. Both acids (oxalic and sulfuric) were mixed at room temperature in an acid tank and diluted to a 5–6 % (w/w) solution using process water. This DA solution was then pumped with a metering type of dosing pump under operating steam pressure. The pretreatment digester can be operated at a temperature range of 140–200 °C, pressure of 4–20 bar, and retention time between 15 and 30 min. The pretreatment reactor is made of SS 316 steel. Pretreatments (low, intermediate, and high-severity acid pretreatment) were conducted with process conditions as discussed in the following sections.

#### Monomeric pretreatment (high-severity mixed-acid treatment)

A batch of 15 kg size reduced dry bagasse (equivalent to 21.4 kg on weight basis) was fed to the pretreatment digester, at a feed rate of 30 kg on dry weight basis per hour, where it was diluted with 29 kg water to a solid consistency of 30 %. This feed was then passed through a plug screw feeder (part of digester system) where 20 kg water was dewatered due to compression. The remaining 30 kg feed was mixed with 20 kg process water (1.3 g H_2_O:g DM) with an acid concentration of 1.5 % sulfuric acid and 1 % oxalic acid on dry weight basis, and the digester was heated to 160 °C for 15 min. Because of the addition of steam and acid solution during pretreatment, the concentration of output solid reached up to 20 % w/w.

#### Mild acid pretreatment (intermediate severity mixed-acid treatment)

Mixed-acid pretreatment was conducted at 180 °C using mixtures of 0.5 % sulfuric acid and 0.5 % oxalic acid on dry bagasse basis with a residence time of 15 min. Other conditions are similar to the one explained in “Monomeric pretreatment (high-severity mixed-acid treatment)”.

#### SE pretreatment (low-severity pretreatment)

SE pretreatment processing was conducted at 180 °C with a residence time of 15 min. Also here, all other details are similar to the standardized format in “Monomeric pretreatment (high-severity mixed-acid treatment)”.

### Post-pretreatment methods and enzymatic hydrolysis

#### Whole-slurry hydrolysis method (Scheme 1)

A batch of 7 kg pretreated slurry obtained from the pretreatment (~20 % w/w total solids) was adjusted to a pH range of 5.2–5.5 using 500 mL 40 % (w/v) NaOH. Then, enzyme hydrolysis experiments were conducted in a 10L reactor equipped with an anchor impeller at 13–14 % total insoluble solids (TIS). Commercial cellulase CTec3 Novozymes enzyme was added to the slurry at 60 mg/g of glucan and was incubated at 50 °C for 120 h. The specific activities of this commercial enzyme are not provided in this study, as it is the proprietary information of the manufacturer. The pH of the enzymatic slurry was maintained in the range of 5.0–5.2. Samples were collected every 24 h for the analysis of sugar and by-products using HPLC (“Compositional analysis methods”). Efficiency of enzymatic hydrolysis was calculated by the ratio of glucose yield (obtained at 120 h after hydrolysis) to the theoretical glucose yield in the solid fraction.

**Fig. 1:**
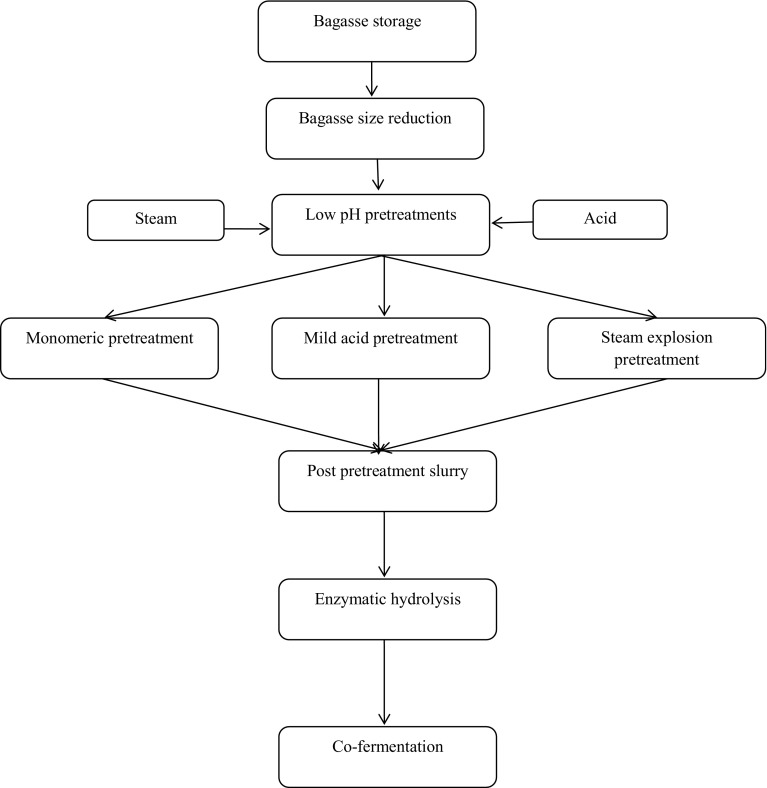
Process flow diagram of whole-slurry hydrolysis (Scheme 1)

Hereafter, this post-pretreatment scheme is referred to as Scheme 1 (Fig. 1) and the mass flow sheet is presented in Figures S1–S3, which provide details of each pretreatment type and mass balances of the entire process.

#### Reslurried hydrolysis methods (Scheme 2)

**Fig. 2:**
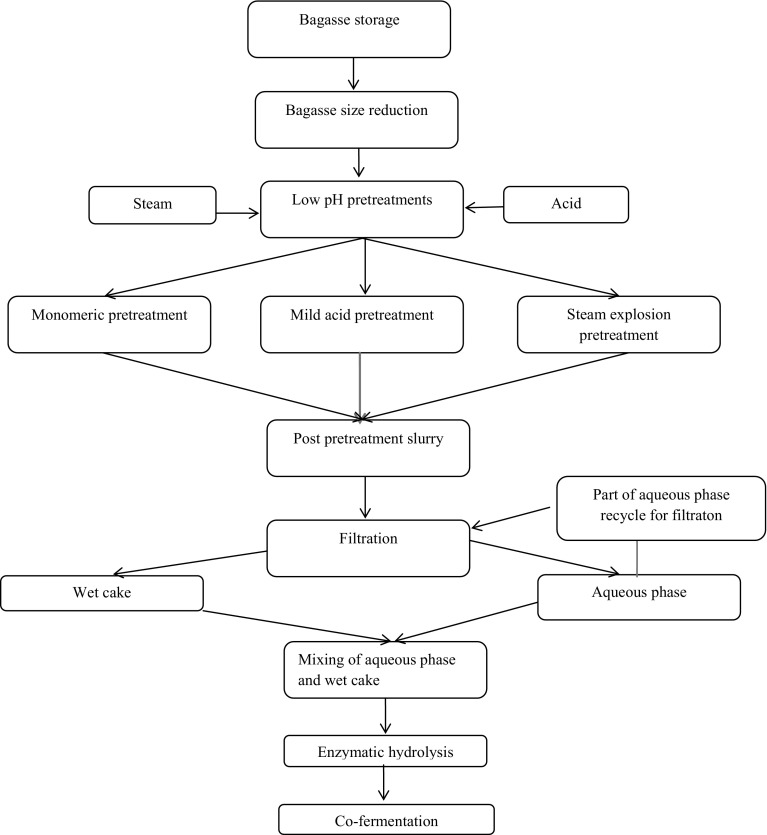
Process flow diagram of reslurry hydrolysis (Scheme 2)

A batch of 70 kg pretreated slurry (~20 % w/w total solids) containing 13–14 % TIS was diluted to 10 % w/w TIS with the addition of 30 kg xylose or xylo-oligomer stream (aqueous) obtained after the initial filtration of pretreated slurry. Initially, some portion of the pretreated slurry was centrifuged to obtain the aqueous phase, which was used for dilution of the TIS to 10 % w/w. This aqueous phase obtained at the start was used for the dilution of the high-solid pretreated slurry. Because of the addition of aqueous phase to the pretreated slurry, a lower concentration of insoluble solids was obtained, enabling effective solid–liquid separation. A filter press with 11 plates (470 × 470 mm) using a hydraulic cylinder serving as a pressing of filter plates provides a filtration pressure of 8–9 bar (g). A filter area of 3 m^2^ and a cake thickness of 30 mm were achieved. After filtration, a solid phase (wet cake) and aqueous phase (xylose-rich stream) were obtained. A small portion of the separated aqueous phase (equivalent to the aqueous phase obtained at the start up of dilution) was reused for the next filtration cycle. The wet cake and the remaining aqueous phase were again mixed to obtain final slurry solids of 20 % (Figs. S4–S6). Following this, enzyme hydrolysis experiments were conducted in a 10L reactor equipped with an anchor impeller at 13–14 % TIS. Commercial cellulase CTec3 enzyme loadings were the same as mentioned in Scheme 1. The enzymatic slurry was incubated at 50 °C for 120 h, while maintaining the pH in the range of 5.0–5.2 using 500 mL 40 % (w/v) NaOH. Samples were collected as described in “Whole-slurry hydrolysis Method (Scheme 1)”. This process scheme is denoted as Scheme 2 (Fig. 2) and the mass flow sheet is presented in Figs. S4–S6.

#### Wet cake hydrolysis (Scheme 3)

**Fig. 3:**
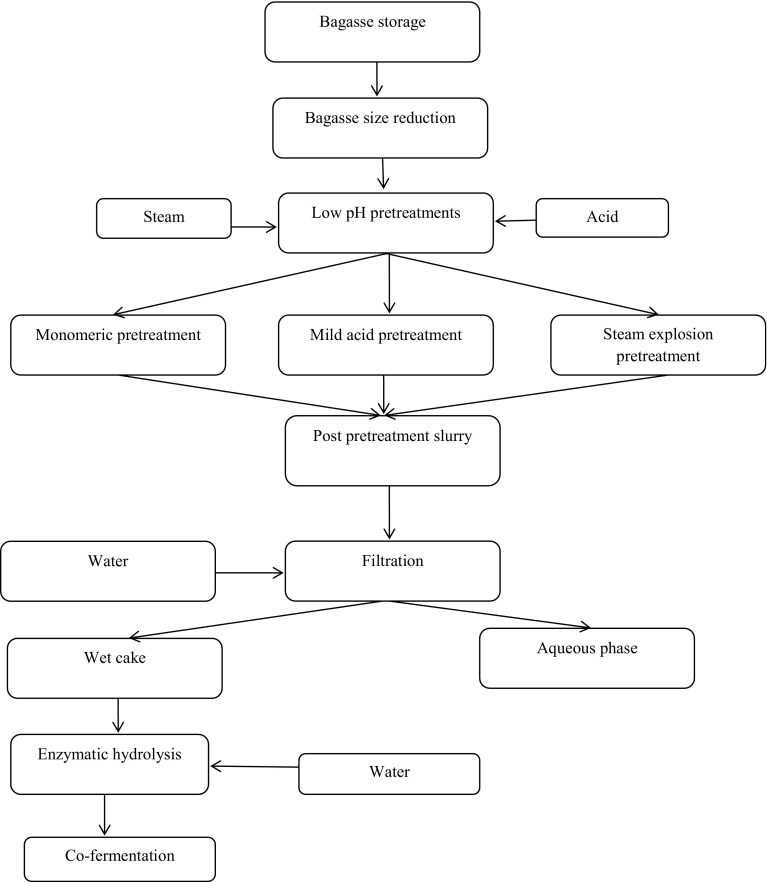
Process flow diagram of wet cake hydrolysis (Scheme 3)

A batch of 70 kg pretreated slurry (~20 % w/w total solids) was diluted with 70 kg fresh water to 5–7 % w/w TIS and then pressure filtered in a filter press, whose parameters are mentioned in “Reslurried hydrolysis Methods (Scheme 2)”. 25 kg solid phase was obtained after separation of pretreated slurry which was further diluted with 37 kg fresh water to a solid consistency of 16–17 % total solids and 14–15 % w/w TIS (Figs. S7–S9). All the enzyme hydrolysis experiments were conducted in a 10L reactor using the commercial cellulase CTec3 enzyme with the same enzyme loading as mentioned earlier. The enzymatic slurry was incubated at 50 °C for 120 h while maintaining the pH in the range of 5.0–5.2 using 220 mL 40 % (w/v) NaOH. Hydrolysis samples were collected every 24 h for the analysis of sugar and by-products using HPLC (“Compositional analysis methods”). This process is referred in this study as Scheme 3 (Fig. 3) and the mass flow sheet is presented in Figs. S7–S9.

### Co-fermentation

Post-enzymatic hydrolyzate from different schemes was further taken for co-fermentation with genetically modified *Saccharomyces cerevisiae*. The yeast was cultured in a yeast extract peptone dextrose (YPD) broth containing yeast extract (10 g/L), peptone (20 g/L), and glucose (30 g/L). The mixture was sterilized at 121 °C for 20 min. Then, a yeast culture preserved in the form of glycerol stock at −80 °C was inoculated onto the broth. The cells were grown in 500-mL Erlenmeyer flasks with 100-mL medium in an orbital shaker at 32 °C and 150 rpm. The pH of the inoculum was adjusted to 5.5 using 40 % w/v NaOH. This inoculum preparation was termed as stage 1. Stage 2 comprising 3 % w/w concentration of fermentable sugars (dextrose sugar source), 1 g/L of diammonium phosphate, and 1 g/L of urea and the mixture was sterilized at 121 °C for 20 min. Following 24-h growth, inoculum from stage 1 was incorporated to stage 2 in 2-L Erlenmeyer flasks with 1-L medium in an orbital shaker at 32 °C and 150 rpm. The pH of the inoculum was adjusted to 5.5 using 40 % w/v NaOH solution. Following 24 h of cell growth, the stage 2 inoculum was transferred to a 10-L reactor containing 9-L enzymatic hydrolyzate (pH 5.5). Additional nutrients, 1 g/L of urea, and 1 g/L of di ammonium phosphate were added to the fermentation medium at 32 °C and 150 rpm using a pitched-blade impeller. The inoculum volume was 10 % of the fermentation medium. A simple flow diagram is depicted in Figure S10.

### Viscosity measurement

Viscosity of monomeric, mild acid, and SE-treated bagasse samples was measured at room temperature using a Brookfield rheometer RHE02000V2.7 with a shear rate of 50 s^−1^. The total solids of around 7–8 % (w/w) were used for viscosity measurements. It was not possible to do measurements at high-solid slurries (>15 % w/w) as the flow was not easy even at higher shear rates. Similar observations have been reported for giant reed (Kadic et al. 2014). Hence, in order to measure viscosity, the pretreated slurry with total solids 20 % w/w was diluted with fresh water to a solid consistency of 7–8 % w/w.

### Dynamic light scattering

Dynamic light scattering (DLS) studies were performed with instrumentation from Brookhaven Instruments Corporation using 90 Plus Particle Sizing Software Version 3.94. Samples (1 % w/w) were dispersed in water by sonication, and the uppermost 1 mL of the dispersion was taken and diluted 100 times for the DLS studies. DLS was performed on all pretreated slurries obtained from different types of pretreatment mentioned earlier.

## Results and discussion

The structurally ordered insoluble biomass constituents can undergo chemical transformation, solubilization, and physical redistribution due to the different pretreatment schemes. The first part of the discussion section focuses on the composition of the solid bagasse, following different severity pretreatments and their effect on the yield of xylose or xylo-oligomers. The second part focuses on the yield of glucose and xylose for the three enzymatic hydrolysis process schemes explored in this study. The combined severity factor is defined as:

$${\text{CSF}} = \log_{10} \left\{ {t{ \times }\exp \frac{{T\text{ - }100}}{14.75}} \right\} - {\text{pH}}$$

in which *t* and *T* are residence time (min) and temperature (°C), respectively. In this work, we have considered three different severities as mentioned in the introduction section.

Similar range of severities was investigated with maple wood and its effect on xylose yields with different types of acids at different acid loadings and temperatures where studied (Zhang et al. 2013).

### Effect of different pretreatments on hemicellulose and glucose yields

The major change due to the dilute mixed-acid pretreatment is the solubilization of hemicellulose (xylose and arabinose) and soluble lignin as phenolics. For all the pretreatments studied, the final total solid of final slurry is 20 % w/w, which includes both insoluble and dissolved solids. The only difference is in the ratio of insoluble solids to dissolved solids depending on the severity of pretreatment. The difference in the compositions of the solid phase post-pretreatment is marginal for the three pretreatments, as xylose is abundantly formed in either oligomeric or monomeric form in the aqueous phase, and hence the residual xylan remaining in the wet cake (2–3 % w/w, Table 1) is always relatively lower. Different patterns of sugar recovery have been observed in the aqueous phase or pre-hydrolyzate (Table 2).

**Table 1:** Carbohydrate and lignin composition (dry basis) of bagasse solids before pretreatment (untreated) and after the following pretreatments: dilute sulfuric acid + oxalic acid (monomeric); dilute sulfuric acid + oxalic acid (mild acid); and steam explosion

Pretreatment type	Glucan (% w/w)	Xylan (% w/w)	Arabinan (% w/w)	Acid-insoluble lignin (% w/w)
Untreated	40.3 ± 2	21.3 ± 0.7	2.2 ± 0.1	19.3 ± 0.8
Monomeric	55.8 ± 2.3	3.1 ± 0.15	0.18 ± 0.03	30.7 ± 1.4
Mild acid	55.8 ± 2.3	3.2 ± 0.16	0.19 ± 0.03	30.1 ± 1.4
Steam explosion	55.7 ± 2.3	3.6 ± 0.17	0.19 ± 0.03	29.3 ± 1.3

**Table 2:** Comparison of sugars and inhibitors produced in the pretreated slurry for the following treatments: dilute sulfuric acid + oxalic acid (monomeric); dilute sulfuric + oxalic acid (mild acid) and steam explosion

Pretreatment	pH	Glucose (% w/w)	Xylose (% w/w)	Xylose oligomer (% w/w)	Acetic acid (% w/w)	HMF (% w/w)^b^	Furfural (% w/w)^b^	Phenolics (% w/w)	Xylanto xylose monomer efficiency (%)	Cellulose to glucose efficiency (%)
Monomeric	1.2	0.60 ± 0.03	4.0 ± 0.2	0.12 ± 0.02	0.42 ± 0.04	0.01	0.01	0.27 ± 0.03	82.8 % ± 2.5	6.5 % ± 0.3
Mild acid	1.8	0.2 ± 0.01	2.4 ± 0.15	1.75 ± 0.1	0.28 ± 0.04	0.01	0.03	0.32 ± 0.03	50 % ± 1.8 (35 ± 1.5)^a^	2 % ± 0.1
Steam explosion	3.5	0.1 ± 0.005	0.78 ± 0.05	3.5 ± 0.2	0.18 ± 0.03	0.02	0.03	0.36 ± 0.04	15 % ± 0.5 (71 ± 2.3)^a^	0.5 % ± 0.05

^a^Efficiency of soluble xylo-oligomers in pretreated slurry

^b^Std. errors for HMF and Furfural are not provided as they are present in very low concentration

Glucose conversion (6.5 %) and monomeric xylose conversion (82.8 %) are highest for monomeric pretreatment (Table 2) with the pH of pretreated slurry at 1.2. The pretreated slurry containing 20 % w/w total solids contains 13 % w/w insoluble solids and the rest 7 % w/w being dissolved solids (Fig. S1). The above recovered mass is equivalent to 650 kg for 1000 kg of dry bagasse. This indicates approximately 35 % solubilization of raw bagasse during the pretreatment. Similar solubilization and xylan recoveries have been reported for the pretreatment of SB using H_2_SO_4_ (2.5 % w/v) at 150 °C for 30 min (Canilha et al. 2011). Concentrations of cellulose and lignin increased on dry basis in the solid phase as compared with the native untreated bagasse (Table 1).

For mild acid pretreatment, monomeric xylose conversion is 50 and 35 % as soluble xylo-oligomers in the slurry, corresponding to a total recovery of 85 % at a pretreated slurry pH of 1.8 (Table 2). Because the acid dosage in mild acid pretreatment is lower as compared to monomeric treatment, the glucose conversion (~2 %) is lower. The solubilized xylo-oligomers can be further hydrolyzed to monomeric xylose using enzymatic treatment or by second-stage acid treatment (Geng et al. 2015; Guo et al. 2012). As an alternative to these methods, fixed-bed reactors packed with a porous solid acid catalyst, such as sulfonic acid resin and organosulfonic acid-functionalized silica catalyst, can also be used for the hydrolysis of soluble xylo-oligomers to monomeric xylose (Kim et al. 2005; Bootsma et al. 2008).The pretreated slurry insoluble solids mild acid treatment is 13.7 % (Fig. S2) and this is equivalent to 685 kg for 1000 kg of dry bagasse used.

In SE pretreatment, 71 % of initial xylan is dissolved as soluble oligomers in the pretreatment media, whereas only 15 % of the initial xylan is recovered in the pretreatment liquid as monomeric xylose, providing overall xylose release of 86 % (Table 2). Glucose monomer conversion in pretreatment is 0.5 % of the initial glucan present in the bagasse. The insoluble solid after SE pretreatment is 14.3 % w/w (Fig. S3) indicating a lesser solubilization of bagasse in the aqueous phase. Previous studies have indicated that, as the acid ratio decreases, the ratio of oligomer to monomer increases, ultimately leading to an increase of pH in pretreated slurries (Zhang et al. 2013; Kootstra et al. 2009).

The formation of inhibitory compounds is reported to be related to the degradation of sugars forming furfural and HMF (Kim et al. 2013). Acetic acid and phenolic compounds are formed from the acetyl groups in hemicelluloses and lignin, respectively. Acetyl groups are also released during the pretreatment, which are present as acetic acid in the pretreated slurry (Kim et al. 2009). Release of acetic acid is highest (0.52 %) in the monomeric treatment and lowest (0.18 %) in the SE pretreatment. This is due to the higher amount of acids added in the monomeric treatment, resulting in a higher monomeric xylose solubilization than steam pretreatment. Phenolics are also released during this pretreatment, leading to solubilization of acid-soluble lignin. Phenolic compounds and other aromatics are formed by pretreatment with an acid catalyst (Martín et al. 2002). However, formation of phenolic compounds is highest for steam pretreatment, because of degradation of more soluble lignin at higher temperatures (Rasmussen et al. 2014).

### Effect of different pretreatment and post-pretreatment process methods on enzymatic hydrolysis efficiencies

Figures 1, 2 and 3 show the overall schematic diagrams of the schemes explored in this study. The effect of these schemes on the yield of enzymatic hydrolysis and ultimately on the concentration of ethanol is discussed in this section. In this study, enzymatic hydrolysis has been performed at almost the same concentration of insoluble solids to ensure consistency, while comparing the efficiency of each process.

#### Whole-slurry hydrolysis scheme (Scheme 1)

In Scheme 1, enzymatic hydrolysis was performed at 13–14 % total insoluble solids (20 % total solids) using Novozymes Cellic CTec3 enzyme at a dose of 60 mg/g glucan content. The combined effect of pretreatment and enzymatic hydrolysis on sugar yields (glucose and xylose) is summarized in Table 3. The monomeric treatment achieved glucan hydrolysis efficiency of 54.5 % and overall xylose efficiency of 85.1 %, including pretreatment and enzyme hydrolysis. Similar glucan hydrolysis efficiency (53.7 %) is observed also for mild acid treatment (Table 3).

**Table 3:** Concentration of sugars and inhibitors before and after enzyme hydrolysis for different pretreatments and different process schemes

Process schemes	Time (h)	Total solids (% w/w)	Total insoluble solids (% w/w)	Glucose (g/L)	Xylose (g/L)	Acetic acid (% w/w)	HMF (% w/w)	Furfural (% w/w)	Phenolics (ppm)	Cellulose to glucose enzymatic efficiency (%)	Overall xylanto xylose efficiency (%) (pretreatment + enzymatic)
Monomeric treatment											
Scheme 1	0	20.1 ± 0.4	13.2 ± 0.3	6.0 ± 0.3	40.1 ± 1.1	0.52 ± 0.04	0.01 ± 0.01	0.01 ± 0.01	0.3 ± 0.03		
Scheme 1	120			50.1 ± 1.5	40.2 ± 1.1	0.54 ± 0.04	0.01 ± 0.01	0.01 ± 0.01	0.3 ± 0.03	54.5 ± 1.7	85.1 ± 2.5
Scheme 2	0	20.2 ± 0.4	13.2 ± 0.3	6.0 ± 0.3	40.3 ± 1.1	0.53 ± 0.04	0.01 ± 0.01	0.01 ± 0.01	0.3 ± 0.03		
Scheme 2	120			60.2 ± 1.6	40.4 ± 1.1	0.55 ± 0.04	0.01 ± 0.01	0.01 ± 0.01	0.3 ± 0.03	65.9 ± 2.0	85.23 ± 2.5
Scheme 3	0	16.3 ± 0.4	14.0 ± 0.3	1.5 ± 0.02	10.1 ± 0.1	0.1 ± 0.01	ND	ND	0.10 ± 0.01		
Scheme 3	120			62.8 ± 1.8	13.5 ± 0.1	0.15 ± 0.01	ND	ND	0.10 ± 0.01	69.0 ± 2.1	84.9 ± 2.5
Mild acid treatment											
Scheme 1	0	19.9 ± 0.4	13.8 ± 0.3			0.28 ± 0.03	0.01 ± 0.01	0.01 ± 0.01	0.32 ± 0.03		
Scheme 1	120			51.1 ± 1.51	39 ± 1.1	0.53 ± 0.05	0.01 ± 0.01	0.01 ± 0.01	0.32 ± 0.03	53.7 ± 1.7	84.9 ± 2.5
Scheme 2	0	20.1 ± 0.6	13.9 ± 0.3	2 ± 0.1	26 ± 0.9	0.29 ± 0.03	0.01 ± 0.01	0.01 ± 0.01	0.33 ± 0.03		
Scheme 2	120			61.1 ± 1.9	39. ± 1.1	0.54 ± 0.05	0.01 ± 0.01	0.01 ± 0.01	0.33 ± 0.03	64.8 ± 2.0	84.1 ± 2.5
Scheme 3	0	17.1 ± 0.4	14.5 ± 0.3	0.9 ± 0.01	3.1 ± 0.05	0.10 ± 0.01	ND	ND	0.09 ± 0.01		
Scheme 3	120			65.3 ± 1.9	5.3 ± 0.1	0.14 ± 0.01	ND	ND	0.09 ± 0.01	69.0 ± 2.1	50.0^a^ ± 1.6
Steam explosion											
Scheme 1	0	20.0 ± 0.5	14.5 ± 0.3	1 ± 0.05	7.8 ± 0.4	0.18 ± 0.02	0.02 ± 0.01	0.02 ± 0.01	0.36 ± 0.04		
Scheme 1	120			43.1 ± 1.2	38.5 ± 1.1	0.56 ± 0.05	0.02 ± 0.01	0.02 ± 0.01	0.36 ± 0.04	48.5 ± 1.6	83.1 ± 2.4
Scheme 2	0	20.1 ± 0.5	14.6 ± 0.3	1 ± 0.05	7.8 ± 0.4	0.18 ± 0.02	0.02 ± 0.01	0.03 ± 0.01	0.36 ± 0.04		
Scheme 2	120			52.2 ± 1.5	40.05 ± 1.1	0.55 ± 0.05	0.02 ± 0.01	0.02 ± 0.01	0.36 ± 0.04	61.9 ± 1.9	83.6 ± 2.4
Scheme 3	0	15.5 ± 0.4	14.2 ± 0.3	0.5 ± 0.01	0.9 ± 0.1	0.08 ± 0.02	ND	ND	0.11 ± 0.1		
Scheme 3	120			60.1 ± 1.7	3.5 ± 0.2	0.55 ± 0.05	ND	ND	0.11 ± 0.1	65.3 ± 2.0	15.0^a^ ± 0.4

^a^Monomeric xylose recovery is lesser as enzyme hydrolysis is conducted in the absence of aqueous phase

Increase of xylose yield from 50 to 85.1 % is observed for mild acid treatment during enzymatic hydrolysis due to xylanase activity of the enzyme cocktail. Um and Van Walsum (2010) showed that enzymes containing hemicellulase could produce up to 84–92 % soluble sugars with either SHF or SSF.

Generation of by-products from pretreatment highly depends on the treatment method used. In this study, high-severity monomeric treatment had the highest acetic acid concentration of 0.52 % at the start of the enzyme hydrolysis, compared with 0.28 % for mild acid treatment and 0.18 % for SE pretreatment. During enzyme hydrolysis, concentration of acetic acid increases from 0.28 to 0.53 % for the mild acid treatment and 0.18–0.56 % for the SE pretreatment. This can be attributed to the conversion of xylo-oligomers to xylose monomers coupled with the release of an acetyl group (Ohgren et al. 2007). Low-pH pretreatment usually degrades some portion of hemicellulosic sugars, forming furan aldehydes (5-HMF and furfural). For mild acid treatment, HMF concentration is found to be 0.01 % (w/w) at the start of enzyme hydrolysis. There is no change in the concentration of furan aldehydes with enzyme hydrolysis.

Lowest glucan hydrolysis conversion is observed for the SE pretreatment. Glucan hydrolysis efficiency of 48.5 % and increase in xylose monomer conversion from 15 to 83.1 % are achieved during enzyme hydrolysis. This very low conversion of glucan is expected as the SE pretreatment is the less severe. Other SE treatment does not have much impact on the biomass other than removing hemicellulose as xylo-oligomers. Another reason for the decrease in enzyme hydrolysis efficiency is the higher amount of soluble xylo-oligomers generated from SE pretreatment (Table 2). Previous studies have indicated that the presence of xylobiose and higher-chain-length xylo-oligomers generated by hydrothermal, acid, or steam pretreatment can inhibit enzyme activity. Therefore, in order to achieve a higher glucan conversion, additional supplementary enzymes have to be added, depending on the feedstock type and composition (Kumar and Wyman 2009). Enzymatic efficiencies of monomeric and mild acid treatments are similar, whereas that of SE treatment is lowest (Table 3).

Release of phenolic compounds from the solubilization of acid-soluble lignin is highest for SE treatment (0.36 %), compared with monomeric pretreatment (0.28 %). These phenolics can impact the degree of hydrolysis efficiencies, leading to decrease in efficiencies (Kim et al. 2009). Both HMF and furfural concentrations were found to be 0.01 % w/w. In general, concentration of these compounds >5 g/L becomes inhibitory for enzyme hydrolysis and affects cellulase enzymes (Cantarella et al. 2004).

The lower enzymatic efficiency of SE pretreatment can also be attributed to the fibrous and solid-like nature of the slurry. Furthermore, the whole-slurry hydrolysis at higher solids has relatively less free water for the enzymes to diffuse to the reaction sites. As a result, enzymes will be crowded at the reaction sites, thus not allowing the process to reaching its full potential (Bommarius et al. 2008). In addition, high viscosity of this slurry prevents an efficient mixing, leading towards lower enzymatic efficiency.

Table 4 shows that the bagasse slurry obtained from SE pretreatment has higher viscosity than that from the other two treatments. As already mentioned “Viscosity measurement”, viscosity measurements were carried out after diluting to 7 % w/w. This allowed us to measure viscosity without problems. Long fiber structure of bagasse provided practical difficulties in viscosity measurements, in particular for bagasse at high-solid loadings. Similar difficulty in estimating viscosity was also observed for giant reed feedstock (Kadic et al. 2014). Viscosity of the slurry for Scheme 1 obtained from monomeric treatment is 0.038 Pa s, whereas that of the steam-exploded bagasse slurry is 0.068 Pa s.

**Table 4:** Comparison of viscosities (Pa s) at 298.15 K and particle sizes for Schemes 1 and 2 prior to enzyme hydrolysis following different pretreatments

	Monomeric pretreatment		Mild acid pretreatment		Steam explosion pretreatment	
	Scheme 1	Scheme 2	Scheme 1	Scheme 2	Scheme 1	Scheme 2
Total solids (% w/w)^a^	7.81	7.82	7.75	7.83	7.92	8.0
Viscosity(Pa s)	0.038	0.016	0.056	0.043	0.068	0.049
Effective diameter (nm)	5222.1	1647.2	14,136.1	5837.1	40,148.4	19,239.2

^a^This total solid reflects the solid used for the measurement of viscosity

In addition to viscosity, particle size affects the enzymatic process. In this regard, DLS experiments were conducted to study the influence of particle size effect. Table 4 shows a comparison of particle sizes of whole slurry and reslurried, pretreated substrates. These measurements were done prior to enzymatic hydrolysis experiments to ascertain the influence of different process schemes on the enzymatic efficiency. It can be inferred from Table 4 that bagasse substrates from Scheme 1 had a higher particle size than those from Scheme 2, regardless of the pretreatment type.

Difference in particle size with pretreatment type is also evident from Table 4. Of the three pretreatment methods studied in this study, monomeric treatment provided the lowest particle size.

#### Reslurried hydrolysis scheme (Scheme 2)

Following Scheme 2, the efficiencies of glucan and xylan hydrolysis after enzyme hydrolysis are 65.9 and 85.2 %, respectively, for monomeric treatment. Similar enzymatic efficiencies have also been obtained for mild acid treatment (Table 3). Similar to Scheme 1, an increase in the concentration of xylose and acetic acid in enzyme hydrolysis is observed for mild acid and SE treatments (Table 3).

Table 3 shows sugar yields, inhibitors, and glucan and xylan hydrolysis efficiencies at the start and at the end of enzyme hydrolysis. Despite having inhibitor profiles similar to Scheme 1 (Table 3), Scheme 2 provides a 10 % higher enzyme hydrolysis efficiency than Scheme 1. This probably indicates that physical restructuring of the bagasse occurring at the mesoscopic and microscopic level following Scheme 2 plays a pronounced role in achieving higher enzyme hydrolysis efficiencies (Eibinger et al. 2014; Adani et al. 2011). Another reason for the increase of efficiency in Scheme 2 is the ease of mixing of slurry at the start of enzyme hydrolysis when the aqueous phase and solids are mixed after separation. The slurry obtained following Scheme 2 shows better flow properties than that obtained following Scheme 1. The pressurized solid–liquid separation and the reslurring process from Scheme 2 might have changed the physical structure and nature of the solids. As a result, higher solids can be effectively mixed during enzymatic hydrolysis.

In Scheme 2, pressure filtration of the pretreated slurry seems to have an impact on the nature of the bagasse. This can be inferred from the viscosity and DLS data. For the monomeric treatment, viscosity of the slurry obtained from Scheme 1 is 0.038 Pa s, whereas that of the slurry obtained from Scheme 2 is 0.016 Pa s (Table 4). DLS data indicate that, for monomeric pretreatment, the particle size following Scheme 2 is 1647.2 nm, compared with 5222.1 nm for those following Scheme 1 (Table 4). This confirms that pressurized filtration induced some physical changes in the bagasse.

Lower particle size enhances available surface area and the penetration of enzymes into porous fiber walls and their subsequent attack on cellulose surface (Peciulyte et al. 2015; Adani et al. 2011). It has been reported in previous studies that decrease of particle size due to post-pretreatment milling increases the accessibility of cellulose enzymes to the substrate and subsequently leads to higher enzyme conversion efficiencies (Chen et al. 2013). On the above basis, it is possible to speculate that pressure filtration used in this study creates new internally accessible surface area, thus enhancing the penetration of enzymes on the cellulose.

Post-pretreatment size reduction is always more beneficial in increasing the enzymatic digestibility than mechanical size reduction prior to pretreatment (Chen et al. 2013) because of its less energy requirement, as some portion of the biomass is already loosened during thermochemical pretreatment (Chen et al. 2013).

SE pretreatment results in lower enzymatic conversions (60 %), the reasons for which have been discussed in “Whole-slurry hydrolysis scheme (Scheme 1)”. The release of xylose into the slurry in enzyme hydrolysis has increased from 15 to 84 %, similar to the result obtained using Scheme 1. This shows that the different process schemes primarily affect the release of glucose by enzymes, and the release of xylose is similar in Schemes 1 and 2. Conversion of cellulose to glucose has been consistently higher for Scheme 2 than Scheme 1 for all three types of pretreatment (Table 3).

#### Wet cake hydrolysis (Scheme 3)

To increase the yield of enzymatic hydrolysis, the pretreated slurries were washed as outlined in Fig. 3, Figs. S7–S9. These washing steps remove maximum soluble inhibitors bound to the substrate and avoid the loss of sugars in the solid residue. In fact, Scheme 3 is the normal routine process used in most of the published articles (Frederick et al. 2013; Hodge et al. 2008). Further, washing of the pretreated biomass will be difficult to replicate at the commercial scale because of its enormous water requirement. Therefore, in order to be cost effective, it is reasonable to minimize or, if possible, avoid the washing process. In this study, the TIS for hydrolysis were kept constant at 14 %. A further increase of 5–7 % in glucan hydrolysis efficiencies is observed, compared with Scheme 2 (Table 3). This can be attributed to the removal of soluble inhibitors bound to the substrate and hence a lower inhibitor concentration at the start of enzyme hydrolysis. The concentrations of acetic acid, furan aldehydes, and phenolics are much lower in Scheme 3 (Table 3) as the inhibitors liberated in the hemicellulose hydrolysis during pretreatment are removed during washing. Minor amount of hemicellulose is still present in the solid fraction after pretreatment. Another rationale is that the pretreatment liquor contains compounds (e.g., solubilized lignin derivatives) that can re-precipitate and recondense on cooling the pretreated biomass, thus inhibiting the cellulase enzymes during the hydrolysis.

For mild acid and steam pretreatments, similar trends of increase in enzymatic efficiencies following Schemes 1 and 2 are also observed (Table 3). The overall xylan to xylose conversion is lower for these pretreatments as the hydrolysis was conducted in the absence of a xylose-rich aqueous stream. An increase in xylose concentration during enzyme hydrolysis is observed for mild acid and steam pretreatments, as some of the soluble xylo-oligomers bound to the substrate are converted to xylose monomers during enzyme hydrolysis (Table 3). In Scheme 3, the aqueous stream separated by filtration is very dilute and will provide very less ethanol concentration post-fermentation. For achieving a higher ethanol concentration, the aqueous stream needs to be concentrated to higher sugar concentrations. However, this may incur an additional cost of evaporation in terms of operating cost and capital cost. In Scheme 3, xylan to xylose monomer conversion is lower for mild acid and steam pretreatments, as soluble xylo-oligomers are formed in the slurry and enzyme hydrolysis was conducted in the absence of an aqueous phase. An additional step of converting the xylo-oligomers to xylose monomers will be required with the help of an acid or enzyme.

### Effect of the different schemes on sugar yield

A noteworthy difference in the process schemes considered in this study is the effect of pressure filtration and solid–liquid separation on pretreatment that can result in an enhanced yield of sugars and consequently ethanol. Although different types of treatment have been explored in this study, the effect of pretreatment is transferred equally to the enzymatic process. This shows that irrespective of the pretreatment type, the different enzymatic hydrolysis schemes used in this study should hold good.

To effectively compare different pretreatment methods and posttreatment process configurations, it is important to provide material balance, enabling the tracking of sugars (cellulose and hemicellulose) in the process for the generation of fermentable sugars. Individual components, focusing on cellulose/glucose, xylan/xylose, and total solids, have been considered for mass balance calculations. Mass balances were established around the process block diagrams shown in Figures S1–S9. Mass balances are based on processing of 1000 kg of dry bagasse. Table 3 summarizes the sugar concentrations of both glucose and xylose after enzyme hydrolysis. Scheme 2 provides a higher sugar concentration and total fermentable sugars than Scheme 1. For the monomeric treatment, Scheme 2 provides total mixed fermentable sugars of 100 g/L and mixed fermentable sugars of 502 kg compared with Scheme 1, which provides 90 g/L and 457 kg, respectively. This difference is mainly due to the lower efficiency of enzyme hydrolysis obtained with Scheme 1 at higher solids. A similar trend is observed for mild acid and steam pretreatments in Scheme 2, thereby achieving a higher sugar concentration and fermentable sugar yield than Scheme 1.

Glucose concentration is highest for Scheme 3, because of higher enzymatic efficiencies achieved by washing the pretreated slurry and hence lower concentration of soluble inhibitors in the enzymatic slurry as shown in Table 3. However, xylose concentration is lower, as the liquid phase is separated after filtration of pretreated slurry. The additional step of washing in Scheme 3 may lead to an increase in the volume of reactors, thus increasing the cost of ethanol production. Washing of pretreated slurry will lead to dilution of sugar in the aqueous stream separated.

For a simple filtration, as done in Scheme 2, with all other things such as solid content constant, the efficiency is higher by 8–10 %. Washing of the pretreated slurry results in a further increase of efficiency by 5–7 %. This shows that filtration mechanism, where pressure filtration is applied, might have affected the ultrastructure of biomass, thereby enhancing the yield of enzymatic hydrolysis.

The efficiency of enzymatic hydrolysis is highest for Scheme 3, although it requires an extra step of washing.

### Co-fermentation of bagasse enzymatic hydrolyzate generated from different schemes

Enzymatic hydrolyzates obtained from all the schemes were taken for co-fermentation trials with a genetically modified co-fermenting *S. cerevisiae* strain with high sugars and tolerance to inhibitors. For Schemes 1 and 2, the initial mixed sugar concentration was in the range of 80–90 g/L, of which 45–55 g/L is glucose and the rest xylose. The consumption of glucose and xylose was not simultaneous, and the former was more preferred. Xylose consumption started only after all glucose was consumed. The total retention time required for the formation of ethanol for Schemes 1 and 2 is 72 h, of which glucose consumption takes about 24 h and xylose consumption takes another 48 h. The rate of consumption of glucose was much higher than that of xylose, which is evident from the lower retention time required for glucose consumption. Table S1 shows the ethanol concentration for different posttreatment schemes explored in this study. Ethanol concentrations are higher for Scheme 2 than Scheme 1, because of the higher enzymatic efficiency. The maximum ethanol production (5.2 % v/v) corresponded to the maximum total sugar released (89.5 g/L) during the enzymatic hydrolysis using Scheme 2 for monomeric treatment. Similar ethanol concentration was also obtained in Scheme 2 for mild acid-treated post-co-fermentation slurry (Table S1).

Post-fermentation, a maximum of 5.1–5.2 % v/v of ethanol, corresponding to theoretical maximum (90 %), is produced from Scheme 2 for monomeric treatment. This indicates that the co-fermenting strain is capable of providing high conversions to ethanol with high sugars and tolerance to inhibitors. This high tolerance could be due to the adaptation of the initial parent strain, which allows the microorganisms to grow in inhibitors and then gradually enhance their tolerance to the inhibitors.

The contribution of initial sugar for Scheme 3 is majorly glucose and less amount of xylose (Table S1). The lower concentration of xylose is attributed to fact that enzyme hydrolysis was conducted in the presence of fresh water. The total retention time for ethanol production was 48 h, of which glucose consumption took 24 h and xylose consumption took 24 h. In Scheme, 3 the retention time required for ethanol conversion is lower, as the initial xylose has a lower concentration at the start of co-fermentation than other Schemes.

## Conclusion

Pretreatment and enzymatic conversion were investigated for different pretreatment and posttreatment process configurations at high TIS concentration. The high-severity dilute mixed-acid treatment resulted in maximum xylose sugars. Of the three post-pretreatment process scenarios investigated, the method which utilized pressure filtration and remixing of liquid with filtered solid achieved more sugar concentration, yield of fermentable mixed sugars, and ethanol concentration. This process scheme also resulted in lower process water for ethanol production from SB. The third scheme, which involved a solid–liquid separation step and extensive washing steps, although, achieved maximum enzymatic efficiency, but at the cost of extra washing, which may be difficult to perform at industrial scale. The use of high solids in the pretreatment (~20 %) using a 1TPD pretreatment pilot plant loaded with unwashed “as received” SB and enzyme hydrolysis at the same solid loading provided reliable data for a commercial scenario. The differences in enzymatic efficiencies between the various schemes studied were attributed to the mechanism of filtration of pretreated slurry, which changes the ultrastructure, making it more amenable for enzymatic hydrolysis to be conducted at high insoluble solids. The use of high-solid model ultimately increases ethanol concentration and reduces energy cost and capital cost associated with distillation and reactors, respectively.

## Electronic supplementary material

Below is the link to the electronic supplementary material.

Supplementary material 1 (DOC 102 kb)

Supplementary material 2 (DOC 47 kb)
