# Supplementary material for: Pretreatment and enzymatic process modification strategies to improve efficiency of sugar production from sugarcane bagasse
Source: 3 Biotech. 2016 Jun 7;6(2):126. doi: 10.1007/s13205-016-0446-2 (PMC4909031; doi:10.1007/s13205-016-0446-2)
Supplement: Supplementary file 1 — Supplementary material 1 (DOC 102 kb) [file 13205_2016_446_MOESM1_ESM.doc]

Sugarcane bagasse (after size reduction)

Quantity - 1000 kg dry

Cellulose - 403 kg

Xylan - 213 kg

**Feed preparation**

Quantity – 1000 kg dry

Total solids - 30% w/w

**Water – 2333 kg**

**Monomeric treatment(Post pretreatment slurry)**

Quantity - 5000 kg

Total solids - 20% w/w

Total insoluble solids -13%w/w

Xylose monomer -210 kg

Unhydrolyzed Cellulose - 375.97 kg

Glucose monomer - 30 kg

Unhydrolyzed xylan - 20.41 kg

Sulfuric acid

(1.5 wt %)

+ oxalic acid

(1 wt %)

Steam

**Post enzymatic hydrolysis slurry (120 h)**

Quantity - 5000 kg

Total solids -20%w/w

Xylose monomer - 202 kg

Glucose monomer - 255.3 kg

Total sugars - 457 kg

**Fermented wash** (72 h)

Quantity – 5373 kg

Ethanol – 4.51%v/v

**Figure S1.**Flow diagram for Scheme 1with mass balance following monomeric (high severity) treatment

**Sugarcane bagasse**

(after size reduction)

Quantity - 1000 kg dry

Cellulose - 403 kg

Xylan - 213 kg

**Feed preparation**

Quantity – 1000 kg dry

Total solids - 30% w/w

**Water – 2333 kg**

Sulfuric acid

**Mild acid treatment (Post pretreatment slurry)**

Quantity - 5000 kg

Total solids - 20% w/w

Total insoluble solids - 13.7%w/w

Xylose monomer -120.98 kg

Xylose oligomers – 84.68

Unhydrolyzed Cellulose – 393.69 kg

Glucose monomer – 10 kg

Unhydrolyzed xylan – 21.92kg

(0.5 wt %)

+ Oxalic

acid (0.5 wt %)

Steam

**Post enzymatic hydrolysis slurry (120 h)**

Quantity - 5000 kg

Total solids -20%w/w

Xylose monomer – 195 kg

Glucose monomer – 244.6 kg

Total sugars – 439.6 kg

**Fermented wash (72 h)**

Quantity – 5371 kg

Ethanol – 4.56%v/v

**Figure S2. Flow** diagram for Scheme 1with mass balance following mild acid (intermediate severity) treatment

Sugarcane bagasse

(after size reduction)

Quantity - 1000 kg dry

Cellulose - 403 kg

Xylan - 213 kg

**Feed preparation**

Quantity – 1000 kg dry

Total solids - 30% w/w

**Water – 2333 kg**

z

**SE(Post pretreatment slurry)**

Quantity - 5000 kg

Total solids - 20% w/w

Total insoluble solids - 14.3%w/w

Xylose monomer -39 kg

Xylose oligomers –172.5 kg

Unhydrolyzed Cellulose –398.49kg

Glucose monomer –5 kg

Unhydrolyzed xylan – 26.24 kg

Steam

**Post enzymatic hydrolysis slurry (120 h)**

Quantity - 5000 kg

Total solids -20%w/w

Xylose monomer –192.5 kg

Glucose monomer –214.5 kg

Total sugars – 407 kg

**Fermented wash (72 h)**

Quantity – 5391 kg

Ethanol – 4.05%v/v

**Figure S3.** Flow diagram for Scheme 1with mass balance following steam explosion treatment

**Sugarcane bagasse**

(after size reduction)

Quantity - 1000 kg dry

Cellulose - 403 kg

Xylan - 213 kg

**Feed preparation**

Quantity – 1000 kg dry

Total solids - 30% w/w

**Water – 2333 kg**

**Monomeric treatment (Post pretreatment slurry)**

Quantity - 5000 kg

Total solids -20% w/w

Xylose monomer -210 kg

Unhydrolyzed Cellulose - 375.97 kg

Glucose monomer - 30 kg

Unhydrolyzed xylan - 20.41 kg

Sulfuric acid

(1.5 wt %)

+ oxalic acid

(1 wt %)

Steam

Recycle of Aqueous phase

Quantity - 1500 kg

Filtration

**Solid phase**

Quantity - 1857 kg

Total insoluble solids - 35%

Cellulose - 375.97 kg

Unhydrolyzed xylan - 20.41 kg

Glucose monomer - 7.24 kg

Xylose monomer - 50.69 kg

Insoluble lignin - 195.63 kg

**Aqueous phase**

Quantity post recycle - 3143 kg

Total solids- 8.7%

Glucose monomer - 22.76kg

Xylose monomer - 159.31kg

**Post enzymatic hydrolysis slurry (120 h)**

Total solids -20% w/w

Glucose monomer -301.26 kg

Xylose monomer - 201 kg

Total sugars - 502.2kg

**Fermented wash(72 h)**

Quantity – 5336 kg

Ethanol – 5.21%v/v

**Figure S4.** Flow diagram for Scheme 2 with mass balance following monomeric

(high severity) treatment

**Sugarcane bagasse**

(after size reduction)

Quantity - 1000 kg dry

Cellulose - 403 kg

Xylan - 213 kg

**Feed preparation**

Quantity – 1000 kg dry

Total solids - 30% w/w

**Water – 2333 kg**

**Mild acid treatment (Post pretreatment slurry)**

Quantity - 5000 kg

Total solids - 20% w/w

Xylose monomer -120.98 kg

Xylose oligomers – 84.68

Unhydrolyzed Cellulose – 393.69 kg

Glucose monomer – 10 kg

Unhydrolyzed xylan – 21.92kg

Sulfuric acid

(0.5 wt %)

+oxalic acid

(0.5 wt %)

Steam

Recycle of Aqueous phase

Quantity – 1850 kg

Filtration

**Solid phase**

Quantity - 1967 kg

Total insoluble solids - 35%

Cellulose – 393.69kg

Unhydrolyzed xylan – 21.92 kg

Glucose monomer -2.55 kg

Xylose monomer - 40.91 kg

Xylose oligomer -24.93

**Aqueous phase**

Quantity post recycle - 3033 kg

Total solids- 8.7%

Glucose monomer - 22.76kg

Xylose monomer – 80.07 kg

Xylose oligomer - 59.75 kg

**Post enzymatic hydrolysis slurry (120 h)**

Total solids -20% w/w

Glucose monomer – 308.8 kg

Xylose monomer – 194 kg

Total sugars - 502.2 kg

**Fermented wash(72 h)**

Quantity – 5345 kg

Ethanol – 5.17%v/v

**Figure S5.** Flow diagram for Scheme 2 with mass balance following mild acid (intermediate severity) treatment

**Sugarcane bagasse**

(after size reduction)

Quantity - 1000 kg dry

Cellulose - 403 kg

Xylan - 213 kg

**Feed preparation**

Quantity – 1000 kg dry

Total solids - 30% w/w

**Water – 2333 kg**

**SE(Post pretreatment slurry)**

Quantity - 5000 kg

Total solids - 20% w/w

Xylose monomer -39 kg

Xylose oligomers –172.5 kg

Unhydrolyzed Cellulose –398.49kg

Glucose monomer –5 kg

Unhydrolyzed xylan – 26.24 kg

Steam

Recycle of Aqueous phase

Quantity –2150 kg

Filtration

**Solid phase**

Quantity – 2042 kg

Total insoluble solids - 35%

Cellulose – 398.49

Unhydrolyzed xylan – 26.24 kg

Glucose monomer -0.92kg

Xylose monomer – 7.3 kg

Xylose oligomer -31.85 kg

**Aqueous phase**

Quantity post recycle - 2958 kg

Total solids-

Glucose monomer – 4.08 kg

Xylose monomer –31.7 kg

Xylose oligomer – 140.65 kg

**Post enzymatic hydrolysis slurry (120 h)**

Total solids -20% w/w

Glucose monomer – 278.8 kg

Xylose monomer – 202.5 kg

Total sugars – 481.3kg

**Fermented wash(72 h)**

Quantity – 5359 kg

Ethanol – 4.64%v/v

**Figure S6.** Flow diagram for Scheme 2 with mass balance following steam explosion treatment

**Sugarcane bagasse**

(after size reduction)

Quantity - 1000 kg dry

Cellulose - 353 kg

Xylan - 213 kg

**Feed preparation**

Quantity – 1000 kg dry

Total solids - 30% w/w

**Water – 2333 kg**

**Monomerictreatment (Postpretreatmentslurry)**

Total solids -20% w/w

Quantity - 5000 kg

Xylose monomer -210 kg

Unhydrolyzed Cellulose - 375.97 kg

Glucose monomer - 30 kg

Unhydrolyzed xylan - 20.41 kg

Sulfuric acid

(1.5 wt %) +

Oxalic acid (1 wt %)

Steam

Fresh water

4285 kg

Filtration

**Solid phase**

Quantity - 1857 kg

Total insoluble solids - 35%

Cellulose - 375.97 kg

Unhydrolyzed xylan - 20.41 kg

Glucose monomer - 7.24 kg

Xylose monomer - 50.69 kg

**Aqueous phase**

Quantity - 7428

Total solids- 4.6%

Glucose monomer - 22.76kg

Xylose monomer - 159.31kg

**Enzymatic hydrolysis (0 hr, before addition of enzyme )**

Quantity - 4669.5 kg

Total solids -15% w/w

Total insoluble solids - 14%w/w

Fresh water

2812.5 kg

**Post enzymatic hydrolysis slurry (120 h)**

Quantity - 4669.5 kg

Total solids -15% w/w

Glucose monomer - 295.1 kg

Xylose monomer - 50.69 kg

Total sugars - 345.8 kg

**Fermented wash(72 h)**

Quantity – 5048

Ethanol – 3.92%v/v

**Figure S7.** Flow diagram for Scheme 3 with mass balance following monomeric (high severity) treatment

**Sugarcane bagasse**

(after size reduction)

Quantity - 1000 kg dry

Cellulose - 353 kg

Xylan - 213 kg

**Feed preparation**

Quantity – 1000 kg dry

Total solids - 30% w/w

**Water – 2333 kg**

**Mild acid treatment (Postpretreatment slurry)**

Quantity - 5000 kg

Total solids - 20% w/w

Xylose monomer -120.98 kg

Xylose oligomers – 84.68 Unhydrolyzed Cellulose –393.69 kg

Glucose monomer – 10 kg

Unhydrolyzed xylan – 21.92kg

Sulfuric acid

(0.5 wt %)

+

Oxalic acid (0.5 wt %)

Steam

Fresh water

4785 kg

**Solid phase**

Quantity - 1967 kg

Total insoluble solids - 35%

Cellulose – 393.69 kg

Unhydrolyzed xylan – 21.92 kg

Glucose monomer -1.27 kg

Xylose monomer – 15.34 kg

Xylose oligomer -10.99 kg

Filtration

**Aqueous phase**

Quantity – 7818 kg

Total solids- 8.7%

Glucose monomer – 8.73 kg

Xylose monomer – 105.64 kg

Xylose oligomer - 73.69 kg

**Enzymatic hydrolysis (0 hr, before addition of enzyme )**

Total solids -15% w/w

Total insoluble solids - 14%w/w

Fresh water

2780.31 kg

**Post enzymatic hydrolysis slurry (120 h)**

Total solids -15% w/w

Glucose monomer – 286.7 kg

Xylose monomer – 25.84 kg

Total sugars – 312.54kg

**Fermented wash(72 h)**

Quantity – 5145

Ethanol –3.56%v/v

**Figure S8.** Flow diagram for Scheme 3 with mass balance following mild acid (intermediate severity) treatment

**Sugarcane bagasse**

(after size reduction)

Quantity - 1000 kg dry

Cellulose - 353 kg

Xylan - 213 kg

**Feed preparation**

Quantity – 1000 kg dry

Total solids - 30% w/w

**SE(Post pretreatment slurry)**

Quantity - 5000 kg

Total solids - 20% w/w

Xylose monomer -39 kg

Xylose oligomers –172.5 kg Unhydrolyzed Cellulose–398.49kg

Glucose monomer –5 kg

Unhydrolyzed xylan – 26.24 kg

Steam

Fresh water

5214.28 kg

**Solid phase**

Quantity - 2042 kg

Total insoluble solids - 35%

Cellulose – 398.49 kg

Unhydrolyzed xylan – 21.92 kg

Glucose monomer -0.53 kg

Xylose monomer – 5.04 kg

Xylose oligomer - 22.29 kg

Filtration

**Aqueous phase**

Quantity – 8172.2 kg

Total solids- 3.5%

Glucose monomer – 4.47 kg

Xylose monomer – 34.96 kg

Xylose oligomer - 150.21 kg

**Enzymatic hydrolysis (0 hr, before addition of enzyme )**

Total solids -15% w/w

Total insoluble solids - 14%w/w

Fresh water

3045 kg

**Fermented wash(72 h)**

Quantity –5515 .1kg

Ethanol – 4.64%v/v

**Post enzymatic hydrolysis slurry (120 h)**

Total solids -15% w/w

Glucose monomer –288.8 kg

Xylose monomer – 5.04 kg

Total sugars – 293.04 kg

**Figure S9.** Flow diagram for Scheme 3 with mass balance following steam explosion treatment

Stage 1

Quantity – 100 mL

Glycerol stock

Yeast Extract

Peptone

Water

Dextrose

Stage 3

Main fermentation

Quantity – 10000 mL

Urea

Water

Post enzymatic slurry

Dextrose source

Urea

Dextrose source

Stage 2

Quantity – 1000 mL

DAP

DAP

Figure S10. Block diagram of Co-fermentation process
