# Supplementary material for: Pretreatment and enzymatic process modification strategies to improve efficiency of sugar production from sugarcane bagasse
Source: 3 Biotech. 2016 Jun 7;6(2):126. doi: 10.1007/s13205-016-0446-2 (PMC4909031; doi:10.1007/s13205-016-0446-2)
Supplement: Supplementary file 2 — Supplementary material 2 (DOC 47 kb) [file 13205_2016_446_MOESM2_ESM.doc]

Table S1. Ethanol and sugars profile of different pretreatments and different process schemes

**Initial glucose (g/L) Initial xylose (g/L) Final Ethanol**

**(% v/v)**

**Residual sugars**

**(g/L)**

**Co-fermentation**

**efficiency (%)**

**Monomerictreatment**

**Scheme1** 44.5±1.1 35.5±0.9 4.51±0.1 1.0±0.1 88.1±2.1

**Scheme2** 53.5±1.5 36.0±0.9 5.21±0.15 0.8±0.1 90.5±2.2

**Scheme3** 55.8±1.6 12.0±0.3 3.92±0.1 0.2±0.05 90.1±2.2

**Mildacidtreatment**

**Scheme1** 45.4±1.1 34.7±0.9 4.56±0.1 0.7±0.1 89.2±2.1

**Scheme2** 54.5±1.5 34.8±0.8 5.17±0.15 0.5±0.1 89.7±2.1

**Scheme3** 57.7±1.5 4.7±0.1 3.57±0.08 0.8±0.1 90.2±2.2

**Steamexplosion pretreatment**

**Scheme1** 38.0±1.0 34.0±0.8 4.05±0.1 0.1±0.05 89.8±2,1

**Scheme2** 46.0±1.1 35.2±0.9 4.64±0.12 0.8±0.1 89.5±2.1

**Scheme3** 53.4±1.4 3.1±0.1 3.26±0.07 0.4±0.08 90.1±2.2
